# Establishment of Standard Methods for *Listeria monocytogenes* Detection from Foods in Japan

**DOI:** 10.14252/foodsafetyfscj.D-25-00023

**Published:** 2026-03-19

**Authors:** Yumiko Okada, Akiko Nakama, Yukako Shimojima, Miki Ida, Hiromi Nakamura, Kayoko Otsuka, Sumi Ebuchi, Akiko Tomaru, Tomoko Nishida, Tomotaka Yoshida, Hideaki Matsuoka

**Affiliations:** 1 National Institute of Health Sciences, 3-25-26, Tonomachi, Kawasaki-ku, Kawasaki, Kanagawa 210-9501, Japan; 2 Tokyo Metropolitan Institute of Public Health, 3-24-1, Hyakunin-cho, Shinjuku-ku, Tokyo 169-0073, Japan; 3 Toyo University, 48-1, Oka, Asaka-shi, Saitama 351-8510, Japan; 4 Osaka Institute of Public Health, 1-3-3, Nakamichi, Higashinari-ku, Osaka-shi, Osaka 537-0025, Japan; 5 Saitama Institute of Public Health, 410-1, Ewai, Yoshimi-machi, Hiki-gun, Saitama 355-0133, Japan; 6 Fukuoka City Institute of Health and Environment, 2-1-34, Jigyouhama, Chuo-ku, Fukuoka-shi, Fukuoka810-0065, Japan; 7 Food Analysis Technology Center SUNATEC, 9-5 Akahorishinmachi, Yokkaichi-shi, Mie 510-0825, Japan; 8 Tokyo University of Agriculture and Technology, 2-24-16, Naka-cho, Koganei-shi, Tokyo 184-8588, Japan

**Keywords:** *Listeria*, standard method, food, Japan

## Abstract

In Japan, several standard methods have been established and published for detecting microbes from foods by “the Committee of the Methods for the Microbiological Examination of Foods (NIHSJ-MMEF)” since 2005. From the results of the Committee’s activities, five of them became official Japanese methods including NIHSJ-08 and NIHSJ-09 which were first published in 2011 and 2014 based on ISO 11290-1 and ISO 11290-2 with some modifications. In this study, a working group consisting of five institutions was established to analyze that the modifications using CHROMagar ^TM^ Listeria was valid; the method developed was applicable for some Japanese foods such as minced tuna; and what the estimated limit of detection at 50% probability (eLOD_50_) were. The eLOD_50_ values were 0.744 CFU/25g for detection from Half-Fraser broth and 1.11 CFU/25 g for detection from Fraser broth, respectively. Finally, this study established and validated the parameters necessary for monitoring food contamination with *Listeria monocytogenes *(*L. monocytogenes*) in Japan.

## 1. Introduction

*Listeria monocytogenes* causes listeriosis, a serious foodborne illness such as bacteremia, meningitis and stillbirth among elders, immune-deficient patients and pregnant women. In Japan, listeriosis is not a mandatory reportable disease hence the actual number of human listeriosis cases is unclear. However, it was estimated to be 0.65 and 1.06 to 1.57 cases per 100,000 people based on the results of an active surveillance conducted between 1996 and 2001^1^^)^ and national database for nosocomial infection and clinical specimen between 2008 and 2011^2^^)^, respectively. Two confirmed cases of listeriosis outbreak caused by natural cheeses in 2001^3^^)^ and poultry meat products in 2022^4^^)^ were reported in the past. The microbiological criterion of *L.monocytogenes* in Japan was established for natural cheeses and uncooked meat products in 2014^5^^)^. The number of *L. monocytogenes* must not exceed 100 CFU/g in five representative samples obtained from the same batch of food. This criterion was determined by the Ministry of Health, Labour and Welfare (MHLW) based on a risk assessment study conducted by Food Safety Commission of Japan in 2013^6^^)^.

Standard test methods for microorganisms in foods have been developed by the Committee of the Methods for the Microbiological Examination of Foods, established in 2005 in Japan^7^^)^, which consists of approximately 20 members, including food microbiologists and Japanese government officials. The standard methods developed by this Committee are based on three policies: international harmonization, establishing culture-based methods, and publicizing the development process. Therefore, most of these standard methods are based on ISO methods or are validated using ISO methods. The Committee has developed 13 standard methods, four technical specifications, and two guidelines. It is currently developing two standard methods, three technical specifications and three guidelines (Table 1) including those for *L. monocytogenes,* which are used as Japanese official methods. Herein, the authors introduce the standard method for detecting and enumerating *L. monocytogenes* in Japan, based on ISO 11290-1:1996^8^^)^, ISO 11290-1:2017^9^^)^, ISO 11290-2: 1998^10^^)^, and ISO 11290-2:2017^11^^)^ with some modifications.

**Table 1.** Methods for detecting/enumerating bacteria in foods by the Committee of MMEF in Japan

Status	NIHSJ	Target organism	Type of method	Finalized	Latest revision	Reference of the official Japanese method	Technical specification
Finalized	01	*Salmonella* spp.	Detection	2009	2019	Yes	
	02	*Campylobacter jejuni/coli*	Detection	2012	2020		
	03	*Staphylococcus aureus*	Enumeration	2009		Yes	
	06	*Vibrio parahaemolyticus*	Detection	2016			
	07	*Vibrio parahaemolyticus*	MPN	2016			
	08	*Listeria monocytogenes*	Detection	2011	2020	Yes	
	09	*Listeria monocytogenes*	Enumeration	2014	2020	Yes	
	15	Enterobacteriaceae	Detection	2011	2020	Yes	
	16	Enterobacteriaceae	Enumeration	2011	2020		
	20	Toxin encoding gene of *Clostridium botulinum*	PCR				✓
	24	*Clostridium perfringens*	Enumeration	2022			
	26	Cereulide	LC-MS/MS	2017			✓
	27	pathogenic *Yersinia enterocolitica*	Detection	2022			
	28	*Bacillus cereus*	Enumeration	2022			
	33	Total cell count for acceptance of raw milk	Microscopic	2023			✓
	34	Guideline for PCR testing	Guideline	2021			✓
	35	*Campylobacter jejuni/coli*	Enumeration	2023			
	38	Guideline for realtime PCR	Guideline	2023			✓
Under construction	29	*Clostridium perfringens*	Detection				✓
	36	Norovirus	Detection				
	39	Guideline for verification	Guideline				
	40	*Listeria* spp.	Detection				✓
	41	*Listeria* spp.	Enumeration				✓
	42	*Clostridium* spp.	Enumeration				

## 2. Methods

### 2.1 Establishment of the *Listeria* Working Group

The Working Group (WG) for *L. monocytogenes* consisted of five institutions: National Institute of Health Sciences (NIHS), Tokyo Metropolitan Institute of Public Health, Saitama Prefectural Institute of Public Health, Osaka City Institute of Public Health and Environmental Sciences (currently Osaka Institute of Public Health), and Fukuoka City Institute of Health and Environment. The study for the first edition of the standard methods for *L. monocytogenes* was conducted from 2008 to 2011.

### 2.2 Stage 1

During stage 1, the WG collected information about the standard methods for *L. monocytogenes* used internationally, such as the International Organization for Standardization (ISO) methods and Bacteriological Analytical Manual in the United States. Finally, the Committee decided the new standard should be based on ISO 11290-1:1996^8^^)^ and 2:1998^10^^)^, which were the latest version at that time, to ensure international harmony.

### 2.3 Stage 2: Modification in Accordance with ISO 11290

#### 2.3.1 Food item verification

For the challenge test, *L. monocytogenes* ATCC 19115 (serotype 4b) was inoculated into food samples at the low and high levels (100-999 CFU/g and 1000-9999 CFU/g, respectively). The inoculum number was counted by direct plating of bacterial suspension onto TSA plates which incubated at 37°C for 48h. An inoculum-free negative control was also prepared. Samples for each level were prepared in triplicate and sent to the WG members as postal packages under the refrigerated condition. During transport, the temperature was recorded by a logger placed with the samples. The minced tuna and cheese samples were prepared at Tokyo Metropolitan Institute of Public Health and NIHS, respectively. The samples containing 10 grams of test portion were kept below 10°C before the tests, which were initiated on the same day in all institutions.

As *L. monocytogenes* can grow at refrigerating temperature, samples exposed to temperatures above 10°C during transportation were excluded from the study. Each sample was suspended in 90 ml of buffered peptone water (BPW, Merck) and homogenized for 1 min in a stomacher. Then, 1 mL of this suspension was spread onto three selective agar plates (about 0.33 mL per plate), and 0.1 ml was inoculated onto two selective agar plates. The inoculated plates containing chromogenic agar, Agar Listeria according to Ottaviani and Agosti (ALOA) (Oxoid, UK) and CHROMagar^TM^ Listeria (CHROMagar, France) were incubated at 37°C ± 1°C for 48 h, and the PALCAM agar plates (Oxoid, UK) were incubated at 30°C ± 1°C for 48 h. After incubation, the number of typical colonies was counted. The statistical analysis of the challenge test for *L. monocytogenes* using two food types was performed with Welch’s t-test using EZR ver. 1.68.

#### 2.3.2 Purification of L. monocytogenes using Tryptic Soy Agar Instead of Tryptic Soy Agar With Yeast Extract

In ISO 11290-1:1996^8^^)^ and 2:1998^9^^)^, Tryptic Soy Agar with yeast extract (TSYEA), consisting of Tryptic Soy Agar (TSA; Beckton Dickinson and Company) supplemented with yeast extract, was used to purify *L. monocytogenes* before the confirmation test. The WG verified that TSA can be used as alternative for TSYEA. Each of five institutions tested three common *Listeria* isolates and two of their own isolates (five isolates per institution; **Table 2**). A single colony from a TSA plate was inoculated into 3 mL of brain heart infusion (BHI) broth (BD) and incubated at 30°C ± 0.5°C for 18 h. After incubation, a loopful of the culture was transferred to a tube containing fresh BHI broth and re-incubated. The culture was serially diluted to obtain adequate colonies on the agar plates. Then, 100 μL of dilution was plated on TSYEA and TSA plates and incubated at 37°C. The colony numbers were recorded after 21, 24, 27 and 48 h.

**Table 2.** Isolates used in this study

	Species	Status	Serotype	Origin	Characteristics	Institution	Isolate used for comparison study	
							TSA/ TSYEA	CAMP/ beta lysin disc
1	*L. monocytogenes*	Common	4b	Chicken meat		A, B, C, D, E	✓*	✓
2	*L. monocytogenes*	Common	3a	Chicken meat	Weak hemolysis, delayed halo formation on chromogenic agar plate	A, B, C, D, E	✓	✓
3	*L. monocytogenes*	Common	1/2a	Chicken meat	Weak hemolysis, delayed halo formation on chromogenic agar plate	A, B, C, D, E	✓	✓
4	*L. monocytogenes*	Unique	1/2b	Raw ham		A	✓	✓
5	*L. monocytogenes*	Unique	1/2b	Ham		A		✓
6	*L. monocytogenes*	Unique	3b	Ham		A		✓
7	*L. monocytogenes*	Unique	1/2c	Cheese		A		✓
8	*L. monocytogenes*	Unique	1/2c	Cheese		A		✓
9	*L. monocytogenes*	Unique	1/2a	Raw ham		A		✓
10	*L. monocytogenes*	Unique	4b	Raw ham		A		✓
11	*L. monocytogenes*	Unique	1/2a	Cheese		A		✓
12	*L. monocytogenes*	Unique	1/2b	Cheese		A		✓
13	*L. monocytogenes*	Unique	4b	Shallot		A		✓
14	*L. monocytogenes*	Unique	4b	Standard strain	ATCC 19115	A	✓	
15	*L. monocytogenes*	Unique	4ab	Chicken meat		B	✓	✓
16	*L. monocytogenes*	Unique	4b	Pickled vegetable		B	✓	✓
17	*L. monocytogenes*	Unique	1/2a	Cheese		B		✓
18	*L. monocytogenes*	Unique	4e	Chicken meat		B		✓
19	*L. monocytogenes*	Unique	1/2c	Chicken meat		B		✓
20	*L. monocytogenes*	Unique	1/2a	Chicken meat		B		✓
21	*L. monocytogenes*	Unique	1/2b	Pickled vegetable		B		✓
22	*L. monocytogenes*	Unique	1/2c	Chicken meat		B		✓
23	*L. monocytogenes*	Unique	1/2b	Pickled vegetable		B		✓
24	*L. monocytogenes*	Unique	4b	Pickled vegetable		B		✓
25	*L. monocytogenes*	Unique	1/2a	Food processing environment		C		✓
26	*L. monocytogenes*	Unique	3a	Salmon		C		✓
27	*L. monocytogenes*	Unique	3b	Salmon		C		✓
28	*L. monocytogenes*	Unique	1/2a	Food processing environment		C		✓
29	*L. monocytogenes*	Unique	1/2b	Smoked salmon		C		✓
30	*L. monocytogenes*	Unique	4b	Smoked salmon		C		✓
31	*L. monocytogenes*	Unique	1/2b	Cod roe		C		✓
32	*L. monocytogenes*	Unique	1/2a	Cheese		C		✓
33	*L. monocytogenes*	Unique	1/2a	Food		C		✓
34	*L. monocytogenes*	Unique	1/2b	Food processing environment		C		✓
35	*L. monocytogenes*	Unique	1/2c	Standard strain	ATCC 7644	C	✓	✓
36	*L. monocytogenes*	Unique	4b	Standard strain	ATCC 19115	C	✓	✓
37	*L. monocytogenes*	Unique	1/2a	Spicy cod roe		D	✓	✓
38	*L. monocytogenes*	Unique	3a	Spicy cod roe		D	✓	✓
39	*L. monocytogenes*	Unique	4b	Standard strain	ATCC 19115	D		✓
40	*L. monocytogenes*	Unique	1/2a	Chicken meat		D		✓
41	*L. monocytogenes*	Unique	1/2b	Spicy cod roe		D		✓
42	*L. monocytogenes*	Unique	1/2b	Food processing environment		D		✓
43	*L. monocytogenes*	Unique	3b	Dairy product		D		✓
44	*L. monocytogenes*	Unique	3c	Spicy cod roe		D		✓
45	*L. monocytogenes*	Unique	4b	Chicken offal		D		✓
46	*L. monocytogenes*	Unique	4b	Minced chicken meat		D		✓
47	*L. monocytogenes*	Unique	4e	Food processing environment		D		✓
48	*L. monocytogenes*	Unique	1/2a	Salami		E	✓	✓
49	*L. monocytogenes*	Unique	4b	Scallop		E	✓	✓
50	*L. monocytogenes*	Unique	1/2a	Patient		E		✓
51	*L. monocytogenes*	Unique	1/2c	Raw ham		E		✓
52	*L. monocytogenes*	Unique	1/2b	Salami		E		✓
53	*L. monocytogenes*	Unique	1/2c	Raw ham		E		✓
54	*L. monocytogenes*	Unique	1/2a	Meat product		E		✓
55	*L. monocytogenes*	Unique	1/2b	Food processing environment		E		✓
56	*L. monocytogenes*	Unique	1/2a	Raw ham		E		✓
57	*L. monocytogenes*	Unique	3b	Salami		E		✓
58	*L. innocua*	Unique		Beef		A		✓
59	*L. innocua*	Unique		Pickled vegetable		B		✓
60	*L. innocua*	Unique		Type strain	ATCC 33090	C		✓
61	*L. innocua*	Unique		Type strain	ATCC 33090	D		✓
62	*L. innocua*	Unique		Pan fried oyster		E		✓
63	*L. ivanovii*	Unique		Unknown		A		✓
64	*L. ivanovii*	Unique		Type strain	ATCC 19119	B		✓
65	*L. ivanovii*	Unique		Food processing environment		C		✓
66	*L. ivanovii*	Unique		Type strain	ATCC 19119	D		✓
67	*L. ivanovii*	Unique		Unknown		E		✓

*Check mark indicates that the checked isolate was used for the comparison study

#### 2.3.3 Hemolysis test using the beta lysin disk instead of the CAMP test

Each of the five institutions used three common and 10-12 unique isolates to examine whether the beta lysin disk test can be an alternative to the confirmatory Christie-Atkins-Munch-Petersen (CAMP) test. The *Listeria innocua* and *Listeria ivanovii* strains used in the beta lysin disk test are shown in **Table 2**. The CAMP test was performed according to ISO 11290-1 using sheep blood agar. The beta lysin disk (Remel) was placed in the center of a separate blood agar plate, and the test strains were inoculated in the adjacent area according to the manufacturer’s instructions. The plate was then incubated at 37°C for 24 h to observe the formation of the hemolytic zone.

### 2.4 eLOD_50_ calculation

To calculate the estimated level of detection at 50% probability (eLOD_50_) of *L. monocytogenes* in Japanese foods by NIHSJ-08:2020, *L. monocytogenes* ATCC19115 (serotype 4b) was inoculated into commercial lightly pickled Chinese cabbage (ingredients: Chinese cabbage, kelp, chili pepper, kelp stock and salt as pickling liquid; pH 4.93; salt concentration 2.1%). The test strain which had been stored in 20% glycerol at −80 °C was used. It was inoculated onto a TSA plate and incubated at 37°C for 24 h. Then, a single colony was inoculated into 4 mL of BHI broth and cultured at 37°C for 18 h. A loopful of the culture was inoculated into 4 mL of BHI broth and incubated at 37°C for 18 h. The bacterial solution was serially diluted in sterile phosphate buffered saline (PBS, 3M) to prepare the low-, medium- and high-dose inoculum. Then, 100 μL of each dose was inoculated into 25 g of pickle sample, which was aseptically weighed and dispensed into a stomacher bag. The same amount of bacterial suspension was inoculated onto two TSA plates, following incubation at 37°C for 24 h to estimate the number of bacteria inoculated. Four samples were prepared for each dose and one for negative control. After inoculation, 225 mL of Half-Fraser broth (Merck Millipore) preheated to 30°C was added and homogenized for 2 min at a stroke of 8.0 in Masticator (IUL, Spain). The initial suspension was incubated for 25 h at 30°C. Then, 0.1 mL of the incubated Half-Fraser broth was inoculated into 10 mL of Fraser broth (Merck Millipore) and cultured for 24 h at 37°C. One loop of incubated Half-Fraser broth was streaked onto ALOA plates and incubated at 37°C for up to 48 h. The same process was repeated for the samples in Fraser broth. Samples that resulted in typical colonies on the selective agar plates were considered positive. The eLOD_50_ values from Half-Fraser broth and Fraser broth, respectively, were calculated using a program available from the ISO website^12^^)^.

## 3. Results

### 3.1 Food Item Verification

Food item verification was performed using Japanese foods to establish the NIHSJ methods for *L. monocytogenes* based on ISO 11290-2:2014 (the former version of 9). The inoculum number of *L. monocytogenes* in the minced tuna ranged from 510 - 520 CFU/g and 5,100 - 5,200 CFU/g at low and high doses, respectively, while that in the cheese ranged from 120 - 150 CFU/g and 1,200 - 1,500 CFU/g at low and high doses, respectively. Although no *L. monocytogenes* was detected from negative control samples, very small numbers of *L. innocua* and *L. ivanovii* were detected in some samples. The suspicious colonies as *Listeria* spp. were detected from negative control samples. They were identified by confirmation tests described in ISO 11290-2:1998. Briefly, the presumptive *Listeria* spp. colonies were tested their abilities of Rhamnose and Xylose utility, hemolysis by CAMP test.

Based on the data of temperature loggers, the cheese samples in one institution were exposed to temperatures above 10°C during transportation and storage before testing. Therefore, the data from this institution were excluded from further analysis. The bacterial counts from each food were converted into logarithms, and the average and standard deviation were calculated for each medium. No differences were observed between the results for tuna and cheese in any of the three media types, either at low or high concentrations (Table 3).

**Table 3.** Results of the comparison study of enumeration of artificially contaminated *L. monocytogenes* from two food matrices (natural cheese and minced tuna) with three types of selective media.

Inoculated dose	Media	t value between tuna and cheese	p value
Low*	ALOA	-0.30726	0.7641
	CHROM***	-1.1457	0.2688
	PALCAM	0.17436	0.8638
High**	ALOA	0.89665	0.3886
	CHROM	1.2047	0.2537
	PALCAM	0.29067	0.7767

* Low dose: 510-520 CFU/g in minced tuna and 120-150 CFU/g in natural cheese.

** High dose: 5100-5200 CFU/g in minced tuna and 1200-1500 CFU/g in natural cheese.

*** CHROMagar^TM^ Listeria

### 3.2 TSA as An Alternative to TSYEA

The colony-forming abilities of three common and two unique isolates on the TSYEA and TSA plates were compared at five institutions. The ratio of the maximum number of colonies on the TSA culture to those on the TSYEA culture varied for common isolate 1, ranging from 0.54 to 1.31 (**Table 4**). The other two common and unique isolates showed a similar tendency, where the colony-forming ability in TSYEA and TSA were not significantly different. This indicates that TSA can be used for purifying putative *L. monocytogenes*.

**Table 4.** Ratios of numbers of typical colony on TSYEA and TSA

4-1. The results of three common isolates in five institutions			
Institution	Ratio for common isolates on TSA/TSYEA		
	Common isolate 1	Common isolate 2	Common isolate 3
A	0.6862745	1.0263158	1.1612903
B	1.3103448	0.8181818	1.4
C	0.9142857	0.5151515	1.3095238
D	1.0357143	1.0294118	1.2432432
E	0.5409836	0.5641026	0.9310345
Ave	0.8975206	0.7906327	1.2090184
SD	0.3006435	0.2452176	0.1783954
Min	0.5409836	0.5151515	0.9310345
Max	1.3103448	1.0294118	1.4
			
4-2. The results of two unique isolates per institution			
Institution	Isolate #	Ratio of unique isolates on TSA/TSYEA	
A	1	0.9125	
	2	1.066666667	
B	1	1.085714286	
	2	1.136363636	
C	1	0.8125	
	2	0.962962963	
D	1	1.032258065	
	2	1.125	
E	1	1.413043478	
	2	0.529411765	
Ave		1.007642086	
SD		0.23159122	
Min		0.529411765	
Max		1.413043478	

Ave: Average of ratio in five institutions.

SD: Standard deviation of ratio in five institutions

Min: Minimum value of ratio for the common isolates

Max: Maximum value of ratio for the common isolates

### 3.3 Beta Lysin Disk Test as An Alternative to the CAMP Test

**Table 5** shows CAMP and beta lysin disk test results performed at five institutions using three common isolates and 10 unique isolates for each institution. While the results for both tests were similar for all unique isolates, those from three institutions indicated that the common isolates 2 and 3 showed weak hemolysis. In two of these institutions, the samples were determined to be hemolysis-negative in the CAMP test and weakly positive in the beta lysin disk test, while the opposite results were observed in the third institution. These results indicate that while the beta lysin disk test results are comparable to those of the CAMP test, while isolates exhibiting weak hemolysis should be carefully analyzed, because the misjudgment of the hemolysis results with weak hemolytic isolates might occur by using both of CAMP test and beta lysin disk test.

**Table 5.** Degree of agreement between the results of CAMP test and beta lysin disc test

	Common isolates (weak hemolysis)		Unique isolates	
Institution	Agree	Disagree	Agree	Disagree
A	3	0	10	0
B	1	2*	10	0
C	3	0	10	0
D	1	2**	10	0
E	1	2***	10	0

* These common isolates were judged as negative for CAMP test and positive for beta lysin disc test.

**These common isolates were judged as negative for CAMP test and positive for beta lysin disc test.

***These common isolates were judged as negative for beta lysin disc test and positive for CAMP test.

### 3.4 Calculation of eLOD_50_

**Table 6** shows the recovery test results of *L. monocytogenes* in lightly pickled Chinese cabbage samples. The measured values ​​of the inoculum were 0.5 CFU/25 g of test portion, 1 CFU/25 g and 8.5 CFU/25 g at low, medium, and high levels, respectively. The eLOD_50_ calculated using the method described in ISO 16140-3:2021^12^^),^^13^^)^ was 0.744 CFU/25 g (one test portion) from Half-Fraser broth, and 1.11 CFU/25 g from Fraser broth. The duration required for the typical colonies differed between cultures from Half-Fraser broth and Fraser broth. When pure bacterial cultures were inoculated onto ALOA plates, typical colony formation was observed within 24 hours (data not shown).

**Table 6.** eLOD_50_ values of NIHSJ-09:2020 from lightly pickled Chinese cabbage with artificially inoculated Lm

Isolation from	Incubation period of selective agar plate (h)	Numbers of Lm-positive sample/numbers of tested sample				eLOD_50_ (CFU/25 g)
		Low dose*	Mid dose*	High dose*	Blank	
Half Fraser broth	24	1/4	0/4	3/4	0/1	-
	48	2/4	2/4	4/4	0/1	0.744
Fraser broth	24	2/4	1/4	4/4	0/1	-
	48	2/4	1/4	4/4	0/1	1.11

* The inoculum numbers were 0.5 CFU/25 g test portion for low dose, 1 CFU/ 25g for medium dose, and 8.5 CFU/ 25g for high dose, respectively

## 4. Discussion

The results conducted by WG confirmed that the enumeration of *L. monocytogenes* in Japanese foods is comparable to that from natural cheese samples. The CAMP test, used to detect hemolysis, can help distinguish *L. monocytogenes* from *L. ivanovii* and *L. innocua*. However, *S. aureus* and *R. equi* strains are also required for this test. As some laboratories find it challenging to maintain these strains, an alternative method which does not use these strains was strongly required. The beta lysin disk test was developed as an alternative to the CAMP test. However, some isolates with weak hemolysis may result in false negative results in CAMP test and beta lysin disk methods. While testing such isolates, a control isolate showing weak hemolysis should be used for quality control. In this study, the beta lysin disk test results were comparable to that of the CAMP test, although caution should be exercised while interpreting the results for isolates with weak hemolysis. After studies by WG, the Committee agreed that stage 3, comprising the full collaborative study, could be skipped because NIHSJ-08 and 09 were based on ISO 11290-1:1996 and 2:1998^8^^,^^10^^)^, Therefore, the final draft was proposed by the WG, and after some editorial modification, the first editions of NIHSJ-08 and 09 were published in 2011 and 2014, respectively^14^^,^^15^^)^. They were revised in 2020 according to the revision of ISO 11290-1 and 2 in 2017^9^^,^^11^^)^. Furthermore, the official Japanese methods for detecting *L. monocytogenes* in natural cheeses and uncooked meat products were changed from the method based on former method by IDF to the methods based on NIHSJ-08 and 09 in 2014. These methods were also revised in 2021.

The current results indicated that the *L. monocytogenes* levels detected in lightly pickled Chinese cabbage using NIHSJ-08:2020 were comparable to those detected in other foods such as vegetables (eLOD_50_=0.6 ± 0.2) and minced meat (eLOD_50_=1.0 ± 0.9)^9^^)^. Further, the results showed the duration required to obtain typical colonies depended on the type of enrichment broth. This phenomenon was not observed in pure bacterial culture (data not shown), suggesting that the food matrices affect the formation of typical colonies. The incubation periods of selective agar plates were for up to 48 h in NIHSJ-08. However, when the plates were incubated beyond 72 h, a white halo was seen around some colonies (data not shown). The pH of the lightly pickled Chinese cabbage used in this study was pH 4.93, which was higher than the pH limit for listerial growth (pH 4.6)^16^^)^. However, as lightly pickled samples might contain other bacteria such as lactic acid bacteria, a longer incubation time might be required for typical colonies formation, as suggested in ISO 11290-1:2017^9^^)^.
